# Dietary and complementary oral supplements for the management of chronic diseases in children: a systematic review

**DOI:** 10.3389/fped.2025.1710200

**Published:** 2026-01-14

**Authors:** Alessio Danilo Inchingolo, Grazia Marinelli, Luisa Limongelli, Francesco Inchingolo, Gianfranco Favia, Laura Ferrante, Angela Di Noia, Cinzia Maspero, Andrea Palermo, Angelo Michele Inchingolo, Gianna Dipalma

**Affiliations:** 1 Department of Interdisciplinary Medicine, University of Bari “Aldo Moro”, Bari, Italy; 2 Department of Biomedical, Surgical and Dental Sciences, Milan University, Milan, Italy; 3 Department of Experimental Medicine, University of Salento, Lecce, Italy

**Keywords:** alternative medicine, children, chronic diseases, dietary supplements, omega-3 fatty acids, probiotics, vitamins

## Abstract

**Aim:**

Chronic diseases in childhood and adolescence represent a growing global challenge, with families often seeking complementary strategies beyond pharmacological treatment. This systematic review aimed to evaluate the efficacy and safety of dietary and oral supplements in pediatric chronic diseases.

**Materials and methods:**

The review was conducted in accordance with PRISMA guidelines. A systematic search of PubMed, Scopus, Web of Science, and Cochrane Library was performed (2005–2025). Eligible studies enrolled children and adolescents (<18 years) with chronic diseases and assessed oral dietary supplements against placebo, standard care, or no intervention. Thirteen studies were included.

**Results:**

The studies investigated autism spectrum disorder (ASD), functional gastrointestinal disorders, cystic fibrosis (CF), type 1 diabetes (T1D), bronchopulmonary dysplasia (BPD) and juvenile idiopathic arthritis. Interventions included probiotics, omega-3/6 fatty acids, vitamins, minerals, glutathione, Kre-Celazine® and Dimercaptosuccinic Acid (DMSA). Most supplements demonstrated measurable bioactivity, such as modulation of the gut microbiota, changes in inflammatory markers, or improvements in functional indices, but clinical benefits were often inconsistent or limited to subgroups. Safety was generally favorable for probiotics, polyunsaturated fatty acids, magnesium, zinc, and vitamin A, whereas DMSA chelation raised significant safety concerns.

**Conclusions:**

Dietary and oral supplements show promise as supportive interventions in pediatric chronic diseases but cannot yet be recommended for systematic clinical use. Larger multicenter trials with longer follow-up, standardized endpoints, and predictive biomarkers are needed to identify responder subgroups and establish evidence-based recommendations.

## 1 Introduction

### 1.1 Chronic diseases in childhood: a growing challenge

Chronic diseases in childhood and adolescence represent an increasingly important challenge for healthcare systems worldwide. Advances in neonatal care and pediatric medicine have significantly improved survival, but these gains have been accompanied by a growing prevalence of chronic conditions such as asthma, cystic fibrosis (CF), type 1 diabetes (T1D), inflammatory bowel disease, and neurodevelopmental disorders including autism spectrum disorder (ASD) (1–6). Collectively, these conditions exert a profound impact on health trajectories, school performance, psychosocial development, and quality of life. Families are often faced with the need for long-term management strategies, which extend beyond pharmacological treatment to encompass lifestyle modifications, nutritional interventions, and supportive care. The economic burden is also considerable, not only for healthcare services but also for caregivers, who must devote substantial time and resources to disease management (7–11).

### 1.2 The role of dietary supplements in pediatric care

Within this broad scenario, attention has increasingly turned to the potential role of dietary supplements and oral complementary interventions. These include a wide spectrum of compounds such as probiotics, vitamins, minerals, omega-3 fatty acids, antioxidants, and plant-derived extracts. The rationale behind their use is multifactorial. First, many chronic pediatric diseases are associated with measurable alterations in nutritional status, immune regulation, or gut microbial composition (12–14). Second, supplements are widely accessible and often perceived as safe by families, who may seek to integrate them into daily care routines. Third, the growing emphasis on personalized medicine has highlighted the potential of nutritional and microbiota-targeted strategies as adjunctive therapies capable of modifying disease courses or symptom expression (15–21).

The microbiota has emerged as a particularly relevant field in this context. A large body of preclinical and clinical evidence suggests that gut microbial composition and diversity play an important role in modulating immune responses, metabolism, and even neurodevelopmental processes (22–27). In children with chronic diseases, dysbiosis is frequently observed, raising the hypothesis that probiotic or prebiotic supplementation could restore a more favorable microbial profile, reduce inflammation, or improve gastrointestinal and systemic outcomes. While such concepts are biologically plausible, clinical translation remains complex, with variable results depending on age, baseline microbiota, disease subtype, and the specific strains administered (28–35).

### 1.3 Vitamins and minerals in chronic diseases

Vitamins and minerals also deserve attention. Deficiencies are common in pediatric chronic conditions due to altered absorption, increased metabolic demand, or dietary restrictions. For example, fat-soluble vitamins may be insufficient in CF, while vitamin D deficiency has been linked to respiratory and autoimmune diseases (36–41). Micronutrients such as zinc and magnesium play key roles in immune defense and metabolic regulation, and their supplementation has been tested as a strategy to improve infection resistance or functional status. Beyond correcting deficiencies, supplementation is also explored for its potential to exert pharmacological effects, such as antioxidant activity, modulation of inflammatory mediators, or support for tissue repair (42–52).

### 1.4 Omega-3 fatty acids and neuro-immune modulation

Omega-3 fatty acids represent another area of interest. Long-chain polyunsaturated fatty acids such as EPA and DHA are critical components of neuronal membranes and exert anti-inflammatory properties (53–60). Their potential role in neurodevelopmental disorders, asthma, inflammatory bowel disease, and other chronic conditions has been extensively studied, with the hope that supplementation could improve behavioral, cognitive, or immunological outcomes. Despite promising mechanistic data, however, clinical evidence in children remains inconsistent, with some studies reporting modest improvements in subgroups but others showing no clear benefit (61–69).

### 1.5 Phytotherapeutic compounds and alternative approaches

Phytotherapeutic compounds, although less systematically investigated in pediatrics, also attract interest as families increasingly turn to plant-based remedies. Herbal preparations, antioxidant blends, and natural extracts are perceived as “gentle” alternatives, but their variability in formulation, dosing, and quality control raises concerns. Moreover, few high-quality pediatric trials exist, leaving major uncertainties regarding both efficacy and safety (70–79).

### 1.6 Clinical relevance and the need for systematic evaluation

Despite this growing interest, significant gaps persist in literature. Evidence is often fragmented, limited to small single-center studies with short follow-up and heterogeneous designs. Outcomes are not standardized, ranging from parent-reported symptom scales to biochemical markers, making cross-trial comparisons difficult. Placebo effects are frequent and sometimes pronounced, particularly in functional disorders, further complicating interpretation. Safety is rarely the primary endpoint, yet it is crucial when interventions are prolonged or involve vulnerable populations such as preterm infants or children with complex comorbidities. In addition, publication bias may exaggerate positive findings, while negative or inconclusive studies remain underreported (80–84). These limitations hinder the formulation of evidence-based recommendations and leave clinicians uncertain about whether, when, and how to advise families regarding supplements. Another critical issue is the lack of stratification by disease subtype or baseline biological profile. Pediatric chronic diseases are highly heterogeneous; for example, not all children with inflammatory bowel disease or ASD exhibit the same microbial or metabolic alterations. Without adequate stratification, potential benefits may be masked by overall negative results (85–92). This highlights the need for precision approaches that identify likely responders based on biomarkers, such as microbiota composition, genetic variants, or nutritional status. To date, only a minority of pediatric trials have incorporated such stratification, and this represents a major research gap (93–96).

The widespread use of supplements in clinical practice further underscores the urgency of systematic evaluation. Surveys indicate that a substantial proportion of families administer dietary supplements to their children with chronic diseases, often without medical supervision. Motivations range from the desire to strengthen immunity to hopes of improving behavioral symptoms or reducing dependence on conventional medications. While the appeal is understandable, unsupervised use raises the risk of interactions, overdosing, or disappointment when expected benefits fail to materialize. Healthcare professionals must therefore be equipped with reliable evidence to guide discussions with families, balancing potential advantages with realistic expectations and known risks.

**Figure 1:**
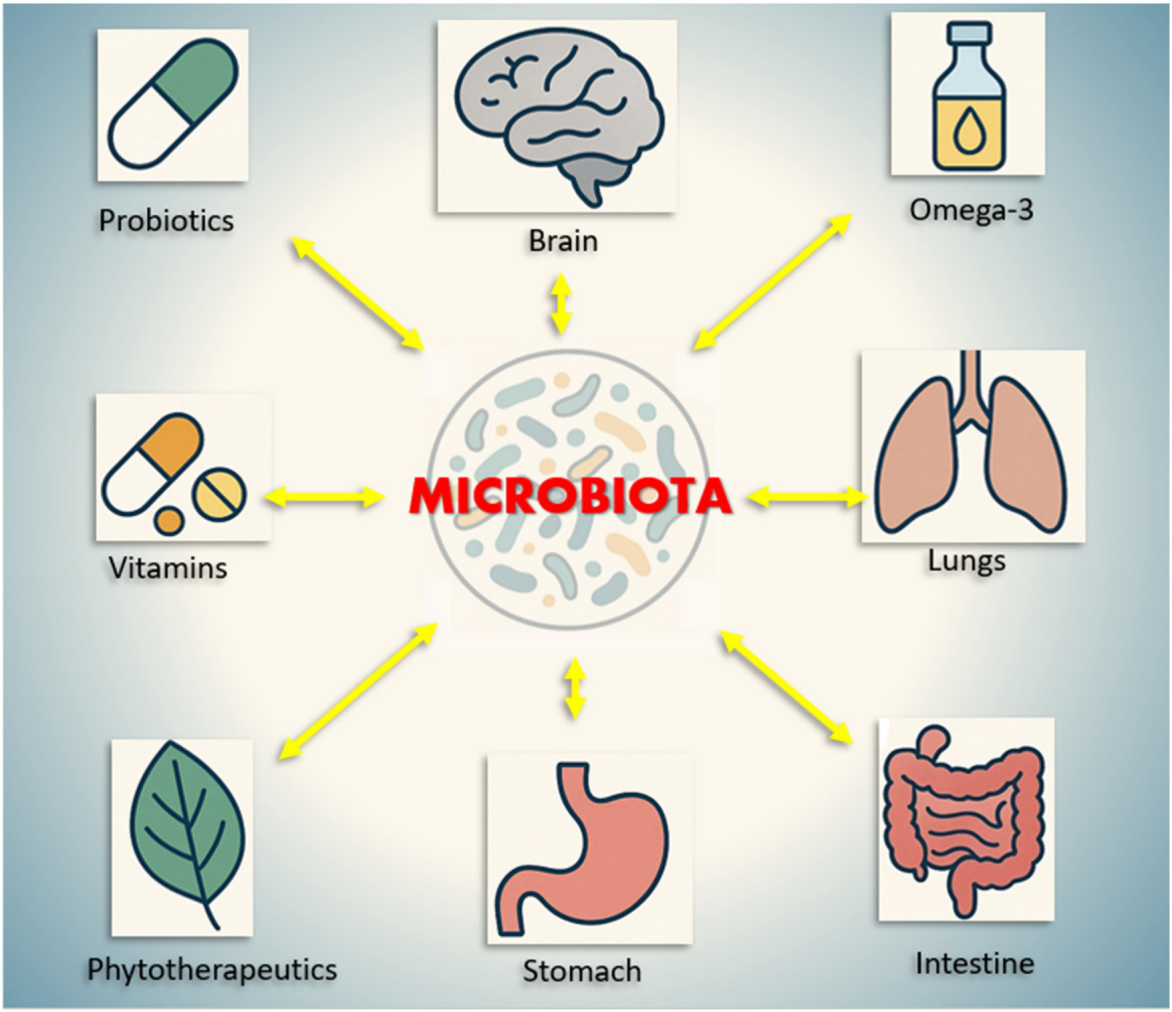
Conceptual illustration of the role of dietary and complementary supplements in pediatric chronic diseases.

A systematic review is therefore warranted to provide a comprehensive synthesis of current knowledge on the efficacy and safety of dietary supplements and oral complementary interventions in pediatric chronic diseases. Beyond compiling the available data, such a review has the potential to clarify which interventions have shown not only measurable biological activity but also reproducible clinical benefits, and under which circumstances these benefits are most evident. At the same time, it allows a critical appraisal of methodological weaknesses that still characterize much of the literature, including small sample sizes, short follow-up periods, and heterogeneous outcome measures, all of which influence the reliability of conclusions. Equally important is the assessment of safety, particularly in vulnerable pediatric populations where long-term supplementation could carry risks that are insufficiently captured in short-term trials (97–103). Finally, by identifying the areas where evidence is scarce or inconsistent, a systematic review can help define priorities for future research, encouraging the design of adequately powered, rigorously controlled studies that focus on clinically meaningful outcomes and pave the way toward a more precise and evidence-based use of supplements in pediatric care. This concept is illustrated in Figure 1.

The objective of the present work is therefore clearly defined: to systematically review the evidence on the efficacy and safety of dietary supplements and oral complementary/alternative interventions in the management of chronic diseases in children. By providing a comprehensive synthesis, this review aims to inform clinical decision-making, support evidence-based counseling of families, and highlight priority areas for future pediatric research. The goal is to clarify the potential role of supplements within integrated care strategies, ensuring that children with chronic conditions receive interventions that are not only biologically plausible but also clinically effective and safe (104, 105).

## 2 Materials and methods

### 2.1 Protocol registration

This systematic review was conducted in accordance with the PRISMA (Preferred Reporting Items for Systematic Reviews and Meta-Analyses) guidelines. The protocol was [registrato/non registrato] on PROSPERO, the International Prospective Register of Systematic Reviews, under the number ID1152059.

### 2.2 Research question (PICO framework)

The central question of this review was formulated according to the PICO framework: “*In children and adolescents with chronic diseases, does the oral administration of dietary supplements or complementary interventions, compared with placebo, no treatment, or standard care, improve clinical outcomes or safety?”.*

In this context, the population considered was pediatric patients up to 18 years of age; the interventions included a wide range of oral supplements, such as probiotics, vitamins, minerals, omega-3 fatty acids and phytotherapeutic compounds; the comparators were either placebo, conventional therapy, or the absence of intervention; and the outcomes focused on both efficacy, measured through clinical and biological parameters—and safety, evaluated through tolerability and reporting of adverse events.

### 2.3 Search strategy

To identify all potentially relevant studies, a comprehensive literature search was carried out in the PubMed, Scopus, Web of Science, and Cochrane Library databases. The search covered the period from January 2005 to January 2025 and was restricted to studies published in English, involving human participants aged between 0 and 18 years. The search strategy combined controlled vocabulary (MeSH terms) and free-text words related to dietary supplements, complementary therapies, and pediatric chronic diseases. In PubMed, for example, the search string included terms such as “*dietary supplements,” “probiotics,” “vitamins,” “omega-3 fatty acids,” “children,” “adolescents,”* and “*chronic disease”*, linked with appropriate Boolean operators.

Overall, the search retrieved 3,274 records: 1,177 from PubMed, 2,059 from Scopus, and 38 from Cochrane.

### 2.4 Study selection

The study selection process was conducted in two stages. First, duplicates were removed, resulting in 2,028 unique records. Titles and abstracts were then screened, leading to the exclusion of 801 reviews and 1,102 articles considered off-topic because they did not involve pediatric populations, or chronic conditions. Thirteen studies met all eligibility criteria and were included in the final synthesis.

The inclusion criteria specified human studies enrolling children or adolescents with chronic diseases and testing oral dietary supplements with clinical or biological outcomes of efficacy and safety. Studies were excluded if they involved only adult populations, investigated parenteral supplementation, were animal or *in vitro* studies, or were available only as conference abstracts, editorials, or narrative reviews. Two reviewers carried out the selection independently, and disagreements were resolved by consensus.

### 2.5 Data extraction and quality assessment

From each eligible study, we extracted the following information: author and year of publication, study design, population and disease investigated, type of supplement and comparator, sample size, treatment duration and follow-up, main outcomes, and key findings. The methodological quality of randomized controlled trials was assessed using the Cochrane Risk of Bias (RoB 2.0) tool, which evaluates domains such as randomization, allocation concealment, blinding, incomplete outcome data, selective reporting, and other potential sources of bias.

## 3 Results

### 3.1 Selected studies and their characteristics

**Figure 2:**
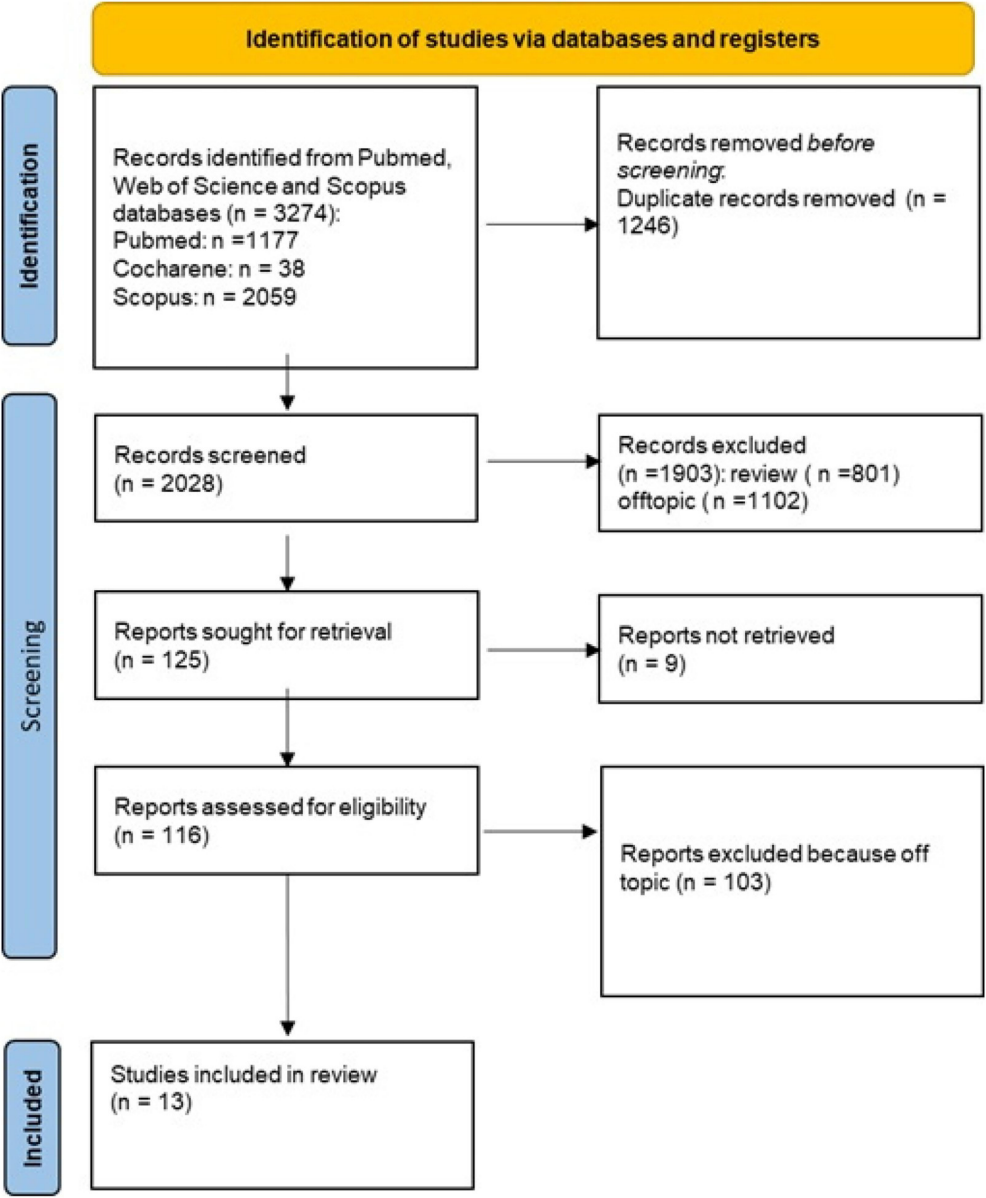
PRISMA flow diagram illustrating the study selection process for systematic review. The chart details the number of records identified, screened, assessed for eligibility, and included in the final analysis, along with reasons for exclusion at each stage.

The database search identified 3,274 records. After removing duplicates and excluding reviews or off-topic articles, 13 studies met the eligibility criteria and were included in the review (Figure 2).

These studies, published between 2008 and 2023, were conducted in various international settings. Most were randomized controlled trials, with some pilot or open-label designs. Sample sizes ranged from small exploratory cohorts to multicenter trials with over 800 participants, and follow-up periods extended from a few weeks to 12 months.

The conditions investigated included ASD, functional gastrointestinal disorders, CF, T1D, bronchopulmonary dysplasia (BPD) in preterm infants, and juvenile idiopathic arthritis. Interventions covered a wide range of oral supplements, most frequently probiotics, followed by omega-3/6 fatty acids, vitamins and minerals (vitamin A, vitamin D, zinc, magnesium), and a few nutraceutical or phytotherapeutic compounds such as glutathione, Kre-Celazine® and Dimercaptosuccinic Acid (DMSA).

Overall, the studies evaluated both efficacy, through clinical and biological outcomes, and safety, mainly in terms of tolerability. A summary of their main features is reported in Table 1.

**Table 1:** Summary of included studies on oral supplements in chronic pediatric diseases.

Authors, Year	Study design	Condition	N (randomized/analyzed)	Age (years)	Intervention (supplement)	Comparator	Treatment & Follow-up	Primary outcome(s)	Main finding(s)
Li et al., (106)	RCT, parallel	Autism Spectrum Disorder (ASD)	∼60/∼55	3–6	Multi-strain probiotics + Applied Behavior Analysis (ABA)	ABA alone	≈3 months	Autism Treatment Evaluation Checklist (ATEC) scores	Greater ATEC improvement and ↑ *Bifidobacterium/Lactobacillus*
Keim et al., (107)	RCT, double-blind	ASD (recent diagnosis)	∼57/∼50	2–<6	Omega-3/6 Fatty Acids (EPA/DHA)	Placebo (canola oil)	≈6 months	Behavior scales	↑ RBC EPA/DHA, ↓ IL-2, no robust behavioral effect
Boone et al., (19)	RCT, double-blind	ASD symptoms in preterm toddlers	∼31/∼28	18–36 months	Omega-3/6 Fatty Acids	Placebo	90 days	Sensory Processing (quadrants)	Medium-large, non-significant effect in sensory sensitivity
Kern et al., (108)	Open-label, non-randomized	ASD	26	3–13	Oral or transdermal glutathione	None	8 weeks	Redox biomarkers	↑ reduced GSH (oral), ↑ cysteine/taurine/sulfates; no clinical benefit
Adams et al., (109)	RCT (chelation)	ASD	65/49	3–8	Oral Dimercaptosuccinic Acid (DMSA) chelation	Placebo	3 × 3-day rounds	Safety, biomarkers	Biochemical changes, no clear clinical benefit, safety concerns
Vázquez-Frías et al., (110)	Multicenter RCT, double-blind	Pediatric Irritable Bowel Syndrome (IBS) (Rome IV)	330	6–18	Bacillus clausii	Placebo	8–16 weeks	Abdominal pain frequency	No superiority vs placebo; secondary benefit in IBS-C subtype
Russo et al., (111)	RCT, double-blind	Functional constipation	∼55	4–12	Probiotic mixture	Placebo	8 weeks	Stool frequency & consistency	Improved bowel parameters with probiotics
Ray et al., (112)	RCT, 12 months	Cystic Fibrosis (CF)	61	2–12	*Lactobacillus rhamnosus* GG (LGG)	Placebo	12 months	Pulmonary exacerbations	No mean benefit; *bifidobacteria*-dominated responders improved
Gontijo-Amaral et al., (113)	RCT, crossover, double-blind	CF	28	6–18	Oral magnesium	Placebo	8 weeks per arm	Clinical & functional indices	Improved respiratory and nutritional indices
Abdulhamid, (114)	RCT, double-blind	CF	32	1–16	Oral zinc	Placebo	6 months	Respiratory infections	Fewer/shorter RTIs with zinc supplementation
Shabani-Mirzaee et al., (97)	RCT, double-blind	Type 1 Diabetes (T1D)	70	6–18	Probiotic mixture	Placebo	90 days	HbA1c	No HbA1c change; ↓ fasting glucose
Rakshasbhuvankar et al., (115)	RCT, double-blind	Preterm infants: Bronchopulmonary Dysplasia (BPD)	807	<28 weeks GA	Enteral vitamin A	Placebo	Until 28 days of life	BPD severity	Suggested reduction in BPD severity depending on dose/timing
Golini et al., (116)	Pilot study, open-label	Juvenile Idiopathic Arthritis	20	6–18	Kre-Celazine® (cetylated Fatty Acids + omega-3)	None	12 weeks	Pain, inflammation scores	Preliminary improvement in pain and inflammation

### 3.2 Quality and risk of bias assessment for the included articles

**Figure 3:**
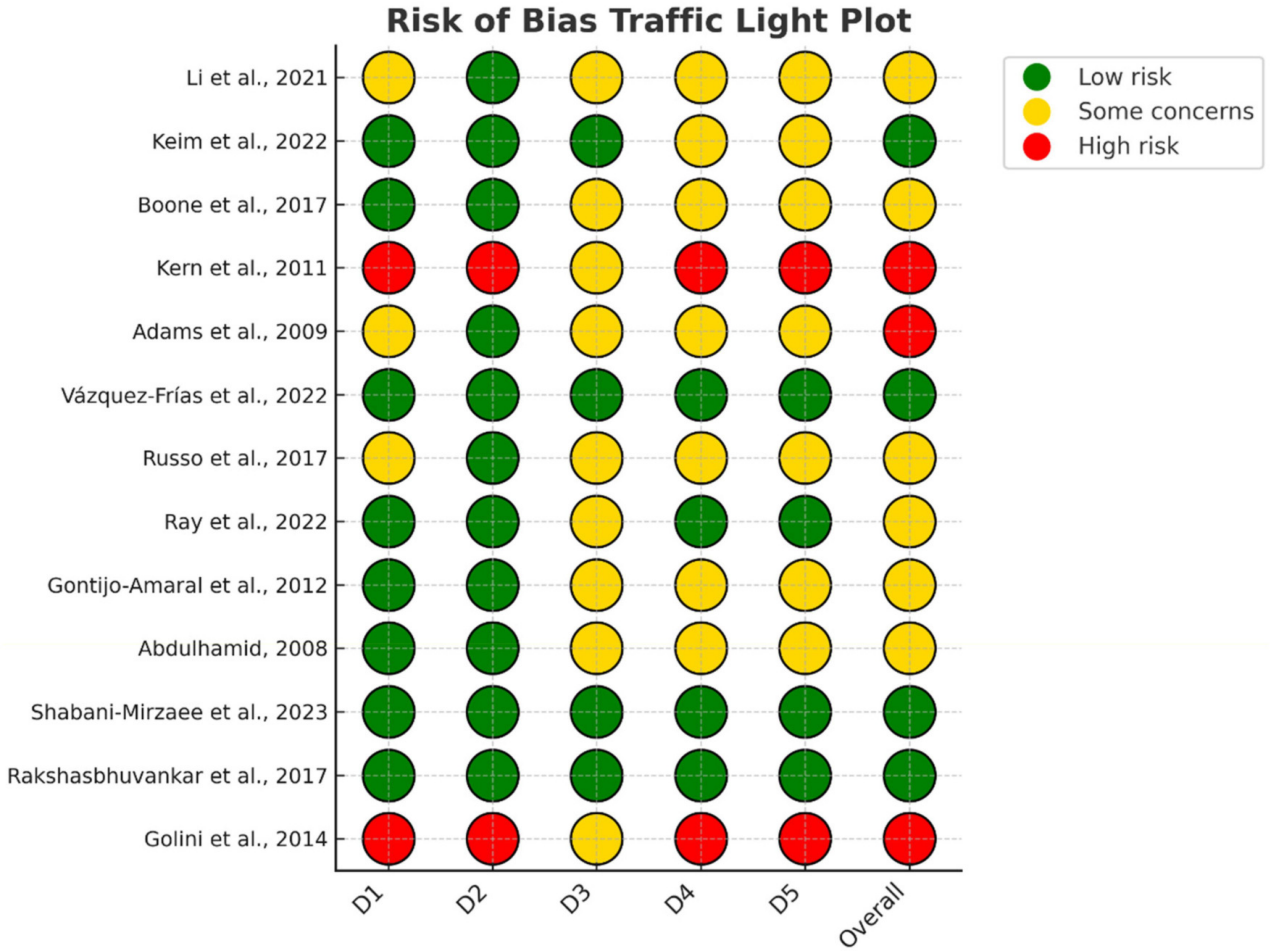
Risk of bias assessment of included studies. Traffic-light plot of individual judgments for each domain and overall risk of bias. Green = low risk; yellow = some concerns; red = high risk.

The overall risk of bias across the thirteen included studies was generally moderate to high, mainly due to common methodological limitations in pediatric supplementation trials. Randomized controlled trials often reported adequate sequence generation and allocation concealment, but details of randomization and blinding procedures were frequently insufficient, limiting the assessment of selection and performance bias. Studies relying on parent-reported outcomes were particularly prone to detection and reporting bias. Other frequent issues included incomplete outcome data, small sample sizes with dropouts, short follow-up durations, and unclear pre-registration or outcome reporting. Open-label designs and underpowered pilot studies further increased the risk of bias. As summarized in Figure 3, these weaknesses indicate that results should be interpreted with caution and highlight the need for future trials with larger samples, longer follow-up, standardized endpoints, and transparent reporting.

**Figure 4:**
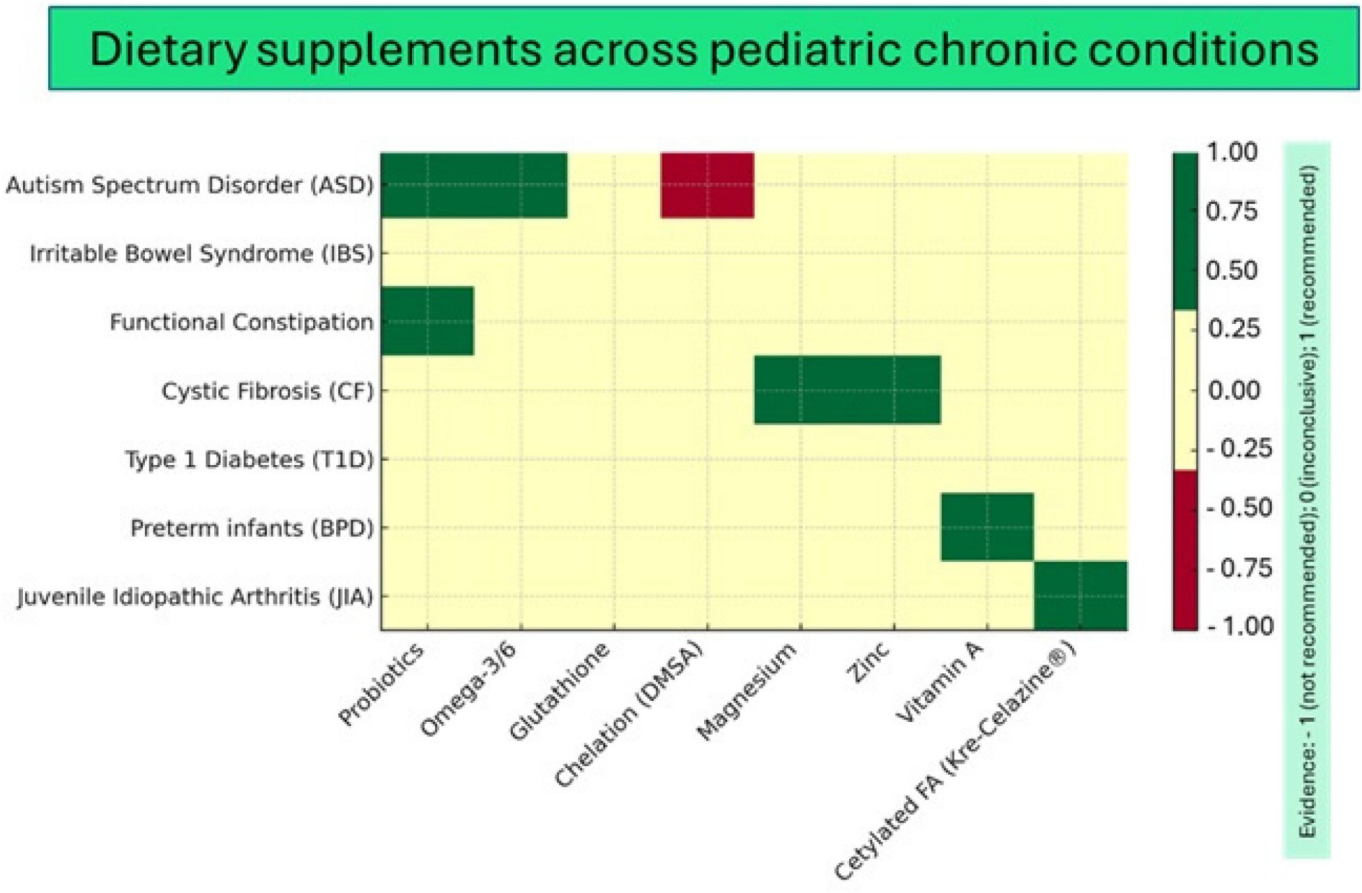
Evidence map of dietary supplements tested in pediatric chronic diseases. Rows represent clinical conditions (ASD, IBS, functional constipation, CF, T1D, BPD in preterm infants, juvenile idiopathic arthritis), while columns represent supplements (probiotics, omega-3/6 fatty acids, glutathione, chelation with DMSA, magnesium, zinc, vitamin A, cetylated fatty acids). Green indicates promising findings, yellow inconclusive evidence, and red interventions not recommended due to safety concerns.

Taken together, the findings of the included trials highlight substantial heterogeneity in interventions, outcomes, and methodological quality. To provide a comprehensive overview of the available evidence across pediatric chronic conditions, an evidence map was generated (Figure 4).

## 4 Discussion

In recent decades, and particularly in recent years, interest in the use of dietary supplements and oral complementary interventions as adjuvants in the management of pediatric chronic diseases has grown steadily across multiple clinical domains, ranging from child neuropsychiatry to functional gastroenterology, pulmonology, pediatric rheumatology, endocrino-metabolic disorders, and neonatology. This interest does not arise in a vacuum but reflects several converging factors: the increasing prevalence of chronic conditions in childhood, the persistent need for additional therapeutic strategies in diseases that rarely have curative treatments, the widespread perception of supplements as safer than conventional drugs, the pressure from families seeking “natural” solutions, and the growing accessibility of these products in the global market. It is therefore essential to carefully analyze the available literature in order to distinguish between documented bioactivity and actual clinical impact, while not neglecting safety concerns and the methodological weaknesses that still limit much of the evidence base (98, 117–123).

One of the most extensively studied areas is ASD, a neurodevelopmental condition characterized by persistent impairments in communication and social interaction, together with restricted and repetitive behaviors, with an estimated prevalence of 1%–2% in Western pediatric populations. The interest in supplements for ASD has been stimulated by several biological observations, including alterations of the gut microbiota, imbalances in oxidative and inflammatory metabolism, and hypotheses of specific nutritional deficiencies. In this context, Li et al. (2021) conducted a randomized trial in preschool children, showing that the addition of a multi-strain probiotic to intensive Applied Behavior Analysis (ABA) therapy was associated with greater improvement in Autism Treatment Evaluation Checklist (ATEC) scores and favorable modulation of fecal composition, with enrichment of *Bifidobacterium* and *Lactobacillus* (106). While methodologically interesting for the integration of a standardized behavioral intervention, the study presented non-negligible limitations, including the small sample size, short follow-up, reliance on parent-reported outcomes, and publication in Chinese, all of which warrant caution in interpretation. Along a similar line, though with a different focus, Keim et al. (2022) investigated the efficacy of omega-3/6 fatty acids in children aged 2 to <6 years with recent ASD diagnosis, documenting a significant increase in RBC EPA/DHA levels and a reduction in IL-2, clear signals of bioavailability and immunological impact, yet without evidence of parallel robust behavioral improvements in the short term (107). Boone et al. (2017), focusing on preterm toddlers with autistic symptomatology, found improvements in both active and placebo groups, but with a non-significant medium-to-large effect size in sensory sensitivity favoring omega-3/6 supplementation, suggesting the possibility of particularly responsive subgroups that merit further investigation in larger studies (19). Kern et al. (2011) explored the antioxidant pathway with glutathione, administered orally or transdermally, documenting biochemical modifications such as increased plasma reduced GSH (oral only) and higher cysteine, taurine, and sulfate levels, without evidence of substantial clinical change (108). The open-label design, lack of placebo control, and predominantly biochemical focus limit the clinical applicability of these findings. Even more controversial, Adams et al. (2009) investigated the safety and efficacy of chelation therapy with DMSA in children with ASD, observing some biochemical effects but no consistent clinical benefit; this approach remains strongly discouraged today in the absence of heavy metal intoxication, due to the risk of essential mineral depletion and systemic adverse effects (109).

Beyond neuropsychiatry, pediatric gastroenterology has represented another fertile ground for supplement research. Irritable bowel syndrome (IBS), a frequent functional condition in childhood, was investigated in a multicenter, double-blind, randomized trial by Vázquez-Frías et al. (2023), which failed to demonstrate superiority of *Bacillus clausii* over placebo at week 8 for the primary endpoint, though secondary signals of efficacy were observed, including reduced abdominal pain at 4 weeks and decreased bloating in the IBS-C subtype at 4 and 16 weeks. It is noteworthy that this trial was conducted during the COVID-19 pandemic and reported an unusually high placebo response rate, probably amplified by behavioral, dietary, and psychosocial changes (110, 124). Also in the gastrointestinal field, Russo et al. (2017) examined the efficacy of a probiotic mixture in functional constipation, showing improvements in stool frequency and consistency, though in a context of considerable heterogeneity of strains, dosages, and endpoints, which complicate comparisons and calls for cautious interpretation (111).

Another paradigmatic condition in which management remains challenging is represented by CF. Ray et al. (2022) conducted a 12-month randomized trial with *Lactobacillus rhamnosus* GG (LGG), reporting that children who developed a “*Bifidobacteria*-dominated” microbiota experienced fewer pulmonary exacerbations, higher FEV1, lower fecal calprotectin, and fewer antibiotic days compared with “*Bacteroides*-dominated” subjects, despite no significant mean benefit in the primary analysis (112, 125, 126). These findings suggest that clinical response may depend less on the intervention itself and more on the induced microbial profile, opening the way to a precision nutrition approach. Regarding micronutrients, Gontijo-Amaral et al. (2012), in a randomized crossover trial, found that oral magnesium supplementation improved some clinical and functional indices, while Abdulhamid (2008) reported a possible reduction in the incidence and duration of respiratory infections with zinc supplementation. Both approaches appear promising but require confirmation through larger and longer studies, since current evidence derives from small samples and short observation periods (113, 114).

From the endocrinology perspective, T1D has been addressed as a chronic condition requiring continuous metabolic control. Shabani-Mirzaee et al. (2023) evaluated probiotic supplementation over 90 days, finding no significant effect on HbA1c but a reduction in fasting glucose (97). While partial, this result suggests a possible complementary role of probiotics in modulating daily glycemic balance, warranting further investigation in longer studies with predefined clinical endpoints such as hypoglycemia frequency, glycemic variability, and insulin requirement. In neonatology, Rakshasbhuvankar et al. (2017) explored whether enteral vitamin A supplementation in preterm infants could reduce the severity of BPD. Interpretation of results, however, must take into account methodological variables such as dosing, timing of administration, co-interventions, and assessment of medium-term respiratory outcomes (115, 127–131). Finally, in pediatric rheumatology, Golini et al. (2014) conducted a pilot study on Kre-Celazine® (cetylated fatty acids plus omega-3) in children with juvenile idiopathic arthritis, reporting potential benefits in pain and inflammation reduction, though within the limits of small sample size and preliminary design, which render conclusions still exploratory (116).

Taken together, these thirteen studies highlight several cross-cutting considerations. On one hand, many supplements demonstrate measurable bioactivity, such as modulation of gut microbiota, increase in long-chain fatty acids in red blood cells, changes in inflammatory cytokines, improvements in respiratory function indices, or reduction of fasting glucose. On the other hand, the translation of these biological signals into robust, reproducible, and generalizable clinical outcomes remains inconsistent or limited to subgroups of patients. Safety appears generally favorable, particularly for probiotics, omega-3/6 fatty acids, magnesium, zinc, and vitamin A within physiological ranges, while controversial approaches such as DMSA chelation carry significant risks and lack support in clinical practice outside clear toxicological indications. Finally, heterogeneity in study design, included populations (often mixing children and adolescents), supplement dosages and formulations, and even comparators (sometimes “active” placebos such as canola oil) complicates meta-analysis and explains divergent results, especially in functional gastroenterology (132–137).

**Figure 5:**
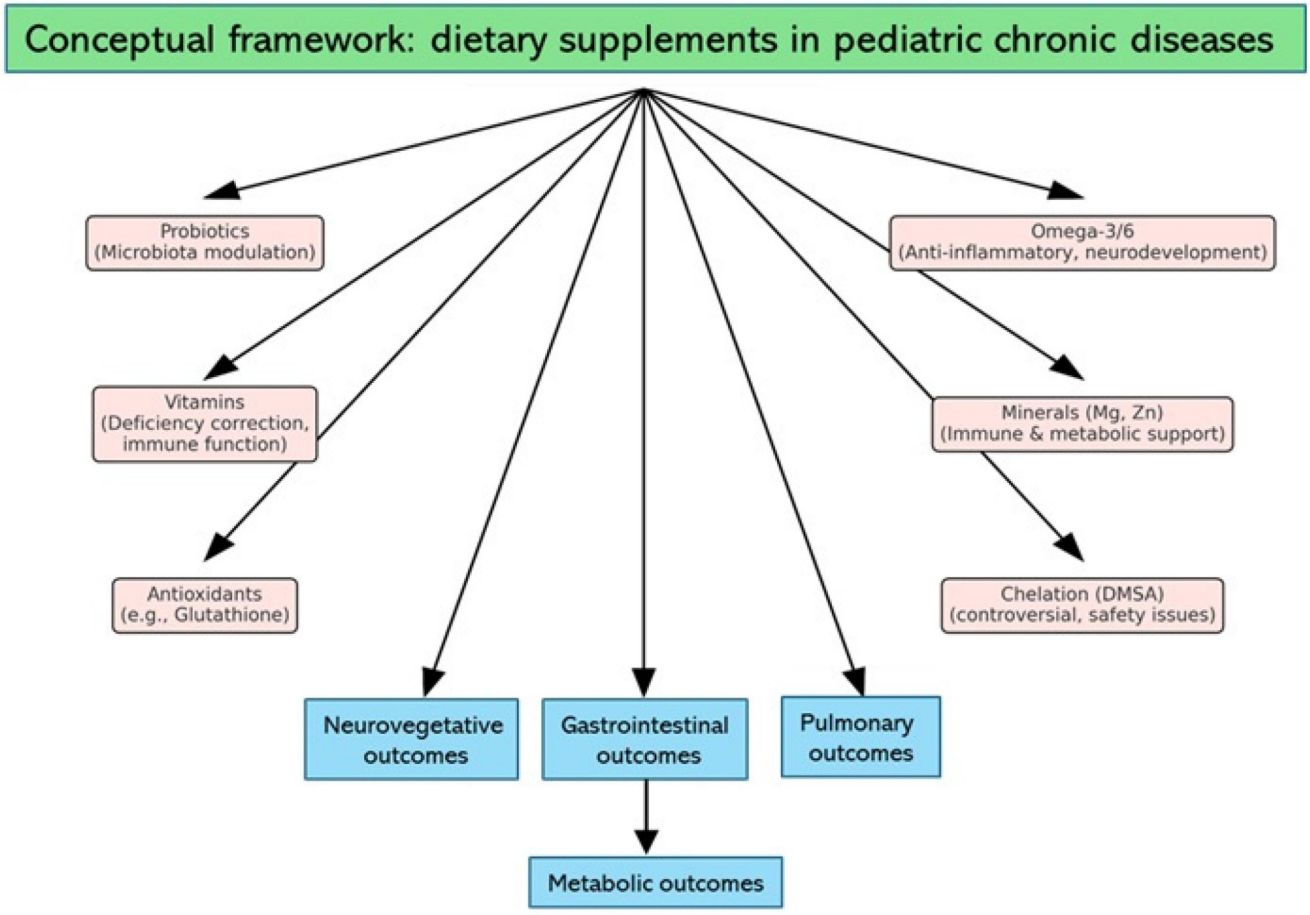
Conceptual framework for dietary supplements in pediatric chronic diseases. Core conditions are potentially modulated by various classes of supplements (probiotics, omega-3/6 fatty acids, vitamins, minerals, antioxidants, chelation). Proposed pathways of action include microbiota modulation, anti-inflammatory effects, correction of deficiencies, and metabolic or immune support, ultimately aiming at improvements in neurocognitive, gastrointestinal, pulmonary, and metabolic outcomes.

This perspective is summarized in a conceptual framework (Figure 5) and in Table 2, outlining how different classes of dietary supplements may act through pathways such as microbiota modulation, anti-inflammatory effects, and metabolic or immune support, with the ultimate goal of improving neurocognitive, gastrointestinal, pulmonary, and metabolic outcomes.

**Table 2:** Oral dietary supplements investigated in pediatric chronic diseases.

Condition	Supplements studied	Main findings
ASD	Probiotics (multi-strain); Omega-3/6 fatty acids; Glutathione; Chelating agents (DMSA)	- Probiotics + ABA: greater improvement in ATEC scores and enrichment of *Bifidobacterium/Lactobacillus.* - Omega-3/6: increased bioavailability and IL-2 reduction; possible subgroup effects on sensory sensitivity. - Glutathione: changes in redox biomarkers without clinical impact. - DMSA: biochemical effects but no consistent clinical benefit.
IBS	Probiotics (*Bacillus clausii*)	No superiority over placebo at week 8; secondary signals in IBS-C subtype (pain, bloating).
Functional Constipation	Probiotic mixtures	Some improvement in stool frequency and consistency.
CF	Probiotics (LGG); Magnesium; Zinc	- Probiotics: *bifidobacteria*-dominated responders had fewer pulmonary exacerbations, better FEV1, less antibiotic use. - Magnesium: improved functional and nutritional parameters. - Zinc: reduced respiratory infections.
T1D	Probiotics	No significant reduction in HbA1c at 90 days; reduction in fasting glucose.
BPD	Vitamin A (enteral)	Suggested reduction in severity of BPD depending on dosing and timing.
Juvenile Idiopathic Arthritis (JIA)	Kre-Celazine® (cetylated fatty acids + omega)	Preliminary evidence of reduced pain and inflammation in a pilot study.
ASD (controversial approach)	DMSA (chelation)	Biochemical changes without robust clinical benefit; safety concerns remain.

### 4.1 Limitations and clinical implications

In light of these findings, the implications for future research are clear. Large, multicenter randomized trials with adequate duration are needed to determine whether the observed biological changes translate into sustainable long-term clinical benefits. The use of predictive biomarkers, ranging from baseline microbiota composition and membrane lipid profiles to micronutritional and genetic status, will be a decisive step toward identifying responder subgroups and advancing true precision nutrition. Equally important will be the adoption of truly inert comparators and standardized, objective clinical endpoints, such as hospitalizations, respiratory exacerbations, pulmonary function tests, and measurable neurocognitive parameters. Cost-effectiveness analyses should also be integrated, considering quality of life, treatment adherence, and family preferences. Only through this approach will it be possible to establish which supplements hold genuine clinical value and which remain confined to biologically interesting but therapeutically inconsequential signals (138–140).

## 5 Conclusions

Dietary and oral supplements represent a promising yet still exploration in the management of pediatric chronic diseases. Current evidence, although preliminary, suggests that in conditions such as ASD, probiotics, polyunsaturated fatty acids, and glutathione may modulate behavioral symptoms, inflammatory parameters, and oxidative metabolism, while in CF, enrichment of the microbiota with *Bifidobacteria* has been associated with better respiratory outcomes and reduced inflammation. By contrast, in children with IBS, results remain uncertain and are often influenced by the high placebo effect and underlying clinical heterogeneity. Overall, these interventions appear generally safe and well tolerated, but the available evidence is not yet sufficient to recommend their systematic use in clinical practice. The heterogeneity of observed responses highlights the need for a personalized approach, in which supplements are not regarded as universal solutions but as complementary tools to be integrated within multidisciplinary care strategies. The challenge for the scientific community will be to translate preliminary signals into validated protocols through large, randomized trials with extended follow-up and the incorporation of predictive biomarkers. Oral supplements in pediatric populations show considerable potential as supportive strategies in the management of chronic diseases, but their use should remain cautious and guided by more robust clinical evidence.

## Data Availability

The original contributions presented in the study are included in the article/Supplementary Material, further inquiries can be directed to the corresponding authors.
